# Stature Estimation From Fragments of Femur in South Indian Population

**DOI:** 10.7759/cureus.78877

**Published:** 2025-02-11

**Authors:** Drakshayini B Kokati, Jayaprakash B.R., Mohammed Jaffer Pinjar

**Affiliations:** 1 Anatomy, KLE Academy of Higher Education &amp; Research's (KAHER's) JGMM Medical College, Hubballi, IND; 2 Anatomy, Government Medical College, Kamareddy, IND; 3 Physiology, All India Institute of Medical Sciences, Deoghar, IND

**Keywords:** femur fragments, forensic anthropology, regression equations, south indian population, stature estimation

## Abstract

Stature estimation is an essential component of forensic anthropology, particularly in identifying individuals in scenarios involving fragmented skeletal remains, such as disasters or legal investigations. This study focuses on deriving regression equations for stature estimation using femoral fragments from the South Indian population. A total of 162 dry femora (96 left and 66 right) were analyzed, measuring parameters including maximum femur length, proximal and distal segments, bicondylar breadth, and various girths. The mean total femur length was 44.31 ± 3.08 cm for left femora and 44.13 ± 2.67 cm for right femora. Pearson correlation analysis revealed a very strong correlation (r ≥ 0.8) with shaft length for both sides, while moderate correlations (r = 0.6-0.8) were observed with parameters such as the proximal segment, girth of the middle shaft, and breadth of the neck. Regression equations were developed for parameters with significant correlations, enabling accurate stature estimation.

The findings underscore the necessity of population-specific data for forensic applications, as skeletal dimensions vary significantly across ethnicities due to genetic, nutritional, and lifestyle factors. The regression model for stature estimation demonstrated high reliability, with R² values reaching 0.91 for shaft length. Additionally, the results align with previous studies on other Indian populations while also highlighting unique regional variations. Despite its strengths, the study is limited by the lack of gender differentiation and demographic diversity, which may affect the generalizability of the findings. These results provide valuable insights for medico-legal investigations and orthopedic applications, offering a robust framework for stature estimation in the South Indian population. Future research should explore advanced statistical techniques and include broader demographic parameters to enhance the precision and applicability of the findings.

## Introduction

Establishing the identity of deceased individuals relies on parameters such as age, sex, race, and stature. Among these, stature is a crucial factor in forensic identification [1]. Stature reconstruction studies are vital for creating identification profiles in forensic applications, allowing comparison with antemortem records to locate missing persons [2]. Anatomists, forensic experts, and anthropologists often provide opinions on skeletal remains as evidence in legal proceedings [3]. In major disasters like plane crashes, excavations, and earthquakes, stature estimation enables the identification of unknown bodies, body parts, and skeletal remains. Regression equations derived from intact long bones are typically used to determine stature across different population groups. However, intact long bones are not always available at disaster sites, necessitating equations based on bone fragments [4].

Human growth and development are influenced by factors such as race, ethnicity, and nutrition, requiring population-specific nomograms. Stature reconstruction using norms from other populations may yield inaccurate results [5]. Long bones from both upper and lower limbs are used to formulate regression equations for stature estimation, with lower limb bones showing a stronger association with body height [6]. The femur, being the longest and strongest bone in the human body, constitutes the greatest proportion of stature. This study focuses on the femur, aiming to provide accurate results. The correlation between stature and long bone length varies across races such as Caucasoid, Negro, and Mongoloid races. Previous studies have established standards for European, American White, and Black races [7].

In India, studies have been conducted on bones like the radius and humerus in the Maharashtra population [8]. Research on different Hindu populations in Orissa, Bihar, and Bengal has shown satisfactory correlations between long bone length and body height [9]. However, body ratios change within specific population groups over time due to lifestyle, diet, and socioeconomic factors [5,6]. Few studies with limited parameters have been documented in the South Indian population [10]. One significant limitation in earlier studies is the lack of population-specific regression equations for stature estimation tailored to the South Indian population. This research uses various parts of the femur for stature estimation, including the length and breadth of different segments, as well as girths of the head, neck, and shaft at different levels. The study aims to reconstruct stature from femur fragments, considering regional factors to address medico-legal issues and assist orthopedics in treating femur-related pathologies and fractures.

## Materials and methods

The study was conducted in adherence to ethical guidelines for the use of human skeletal remains in research. Approval was obtained from the Institutional Ethics Committee (IEC) of Shimoga Institute of Medical Sciences, Karnataka, under the approval number SIMS/PG/S/IEC/11/2011-2012. The research followed all ethical protocols to ensure the responsible and respectful use of human skeletal material.

The study utilized dry and processed femora from both sides, regardless of gender, aged between 30 and 60 years based on the ossification status of the femora, sourced from the osteology sections of the Department of Anatomy and Forensic Medicine, Shimoga Institute of Medical Sciences, Karnataka. Conducted as a cross-sectional prospective study over a three-year period from December 2011 to January 2014, a convenient sampling technique was employed to collect a total of 162 femora, comprising 96 from the right side and 66 from the left, all randomly selected. The research aimed to establish regression equations for stature estimation using femoral fragments.

The inclusion criteria for the study were dry, fully ossified femora of individuals aged between 30 and 60 years, free from visible deformities or abnormalities. Bones from both sexes were included in the analysis. The exclusion criteria ruled out femora that were diseased, damaged, or broken, as well as those with visible signs of pathological conditions, deformities, or incomplete ossification. These criteria ensured that only intact and healthy specimens were used for accurate measurements and analysis.

The materials required for the research included spreading calipers, vernier calipers, a non-elastic measuring tape, and an osteometric board. All measurements were taken three times to ensure accuracy, and the mean value was recorded. Bone length was measured in centimeters, while fragment lengths and girths were recorded in millimeters. Measurements were taken using an osteometric board for the maximum femur length, vernier calipers for femur fragments, and a measuring tape for bone girths [11]. The specific parameters measured included total femur length, proximal segment length, two distal segment lengths, bicondylar/lower epiphyseal breadth (BCB), girths of the upper, middle, and lower shafts, girths of the head and neck, breadth of the neck, medial condylar breadth (MCB), and lateral condylar breadth (LCB) [11-13].

Descriptive statistics were calculated for all measurements of left and right femoral fragments. Data normality was tested using the Shapiro-Wilk test. Pearson correlation coefficients were used to assess the relationship between femoral fragments and total femur length for both sides. Simple linear regression equations were formulated for parameters that demonstrated significant correlations. Additionally, constants, regression coefficients, standard errors of estimate, and p-values with confidence limits were calculated for each parameter. Statistical significance was set at p <0.05, and all analyses were performed using the latest SPSS software version 20.0 (IBM Corp., Armonk, NY).

## Results

Table 1 shows the number, minimum, maximum, mean, and standard deviation of fragments of femora on both sides.

**Table 1:** Measurements of fragments of left and right femora MCB, medial condylar breadth; LCB, lateral condylar breadth.

Parameter	Left femora				Right femora			
	N	Minimum (cm)	Maximum (cm)	Mean ± SD	N	Minimum (cm)	Maximum (cm)	Mean ± SD
Total length	96	38	55	44.31 ± 3.08	66	39	48	44.13 ± 2.67
Proximal segment	96	6.00	9.65	7.58 ± 0.67	66	6.0	8.5	7.74 ± 0.65
Distal segment 1	96	7.8	11.3	8.81 ± 0.82	66	7.50	10.37	8.57 ± 0.85
Distal segment 2	96	3.4	5.0	4.29 ± 0.31	66	3.49	4.77	4.20 ± 0.35
Bicondylar/Lower epiphyseal breadth	96	6.5	8.5	7.51 ± 0.52	66	6.5	8.8	7.70 ± 0.51
Shaft length	96	30	45	35.41 ± 2.67	66	30	40	35.21 ± 2.32
Girth of the upper shaft	96	8	11	9.09 ± 0.82	66	8	10	9.13 ± 0.64
Girth of the middle shaft	96	7	10	8.40 ± 0.6	66	8	10	8.55 ± 0.63
Girth of the lower shaft	96	8.0	11.0	9.02 ± 0.74	66	8	10	9.08 ± 0.7
Girth of the head	96	12	17	13.68 ± 1.05	66	12	16	14.07 ± 1.1
Girth of the neck	96	9.0	12.0	10.39 ± 0.76	66	9.0	12.5	10.71 ± 0.86
Breadth of the neck	96	2.50	4.00	3.18 ± 0.37	66	2.28	4.00	3.31 ± 0.38
MCB	96	5	7	5.82 ± 0.41	66	4.27	6.50	5.56 ± 0.52
LCB	96	4.84	6.77	5.77 ± 0.44	66	5.00	6.65	5.97 ± 0.44

Table 2 shows the Pearson correlation coefficient of fragments with total length of left and right femora and p-values. On analysis, a very strong correlation (r = at least 0.8) was seen with shaft length for both the left and right femur. The proximal segment, the girth of the middle shaft, the girth of the lower shaft, the girth of the head, the breadth of the neck, and LCB showed moderately strong correlation (r = 0.6-0.8) for both left and right femur. Distal segment 2 showed a moderately strong correlation (r = 0.6-0.8) for the left femur, but the same segment showed a fair correlation (r = 0.3-0.5) for the right femur. Distal segment 1 showed a moderately strong correlation (r = 0.6-0.8) for the right femur, but the same segment showed a fair correlation (r = 0.3-0.5) for the left femur. BCB, the girth of the upper shaft, the girth of the neck, and MCB showed a moderately strong correlation (r = 0.6-0.8) for the left femur, whereas the same parameters showed fair correlation (r = 0.3-0.5) for the right femur.

**Table 2:** Pearson correlation coefficient of fragments with total length of femora and p-values MCB, medial condylar breadth; LCB, lateral condylar breadth. **Correlation is significant at the 0.01 level (two-tailed).

Parameter	Left femora		Right femora	
	r-Value	p-Value	r-Value	p-Value
Proximal segment	0.682**	<0.001	0.683**	<0.001
Distal segment 1	0.438**		0.636**	
Distal segment 2	0.672**	<0.001	0.447**	<0.001
Bicondylar/Lower epiphyseal breadth	0.601**		0.579**	
Shaft length	0.953**	<0.001	0.920**	<0.001
Girth of the upper shaft	0.732**		0.420**	
Girth of the middle shaft	0.775**	<0.001	0.628**	<0.001
Girth of the lower shaft	0.755**		0.646**	
Girth of the head	0.697**	<0.001	0.705**	<0.001
Girth of the neck	0.697**		0.552**	
Breadth of the neck	0.665**	<0.001	0.727**	<0.001
MCB	0.708**		0.572**	
LCB	0.657**	<0.001	0.698**	<0.001

Simple linear regression equations

Tables 3, 4 show the constant, regression coefficient, standard error of estimate, and p-value with confidence limits from fragments of left and right femora, respectively.

**Table 3:** Constant, regression coefficient, standard error of estimate, and p-value with confidence limits from fragments of left femora RCOE, regression coefficient; SE, standard error of estimate; LCI, lower confidence interval; UCI, upper confidence interval; MCB, medial condylar breadth; LCB, lateral condylar breadth.

Parameter	Constant	RCOE	SEE	p-Value	95% LCI	95% UCI	R^2^
Proximal segment	20.53	3.14	0.35	<0.001	2.45	3.83	0.47
Distal segment 2	15.97	6.6	0.75	<0.001	5.11	8.09	0.45
Bicondylar/Lower epiphyseal breadth	17.40	3.58	0.49	<0.001	2.61	4.56	0.36
Shaft length	5.39	1.1	0.04	<0.001	1.03	1.17	0.91
Girth of the upper shaft	19.31	2.75	0.27	<0.001	2.23	3.27	0.54
Girth of the middle shaft	10.73	4.00	0.34	<0.001	3.33	4.67	0.60
Girth of the lower shaft	16.09	3.13	0.28	<0.001	2.57	3.68	0.57
Girth of the head	16.47	2.04	0.22	<0.001	1.61	2.47	0.48
Girth of the neck	14.85	2.84	0.30	<0.001	2.24	3.43	0.49
Breadth of the neck	26.55	5.59	0.65	<0.001	4.31	6.88	0.44
MCB	13.63	5.27	0.54	<0.001	4.19	6.35	0.50
LCB	17.92	4.6	0.54	<0.001	3.50	5.65	0.43

**Table 4:** Constant, regression coefficient, standard error of estimate, and p-value with confidence limits from fragments of right femora RCOE, regression coefficient; SE, standard error of estimate; LCI, lower confidence interval; UCI, upper confidence interval; MCB, medial condylar breadth; LCB, lateral condylar breadth.

Parameter	Constant	RCOE	SEE	p-Value	95% LCI	95% UCI	R^2^
Proximal segment	22.59	2.78	0.37	<0.001	2.04	3.52	0.47
Distal segment 2	29.85	3.39	0.85	<0.001	1.7	5.09	0.19
Bicondylar/Lower epiphyseal breadth	20.88	3.02	0.53	<0.001	1.96	4.08	0.33
Shaft length	6.79	1.06	0.06	<0.001	0.95	1.17	0.85
Girth of the upper shaft	28.08	1.76	0.47	<0.001	0.81	2.70	0.18
Girth of the middle shaft	21.45	2.65	0.41	<0.001	1.83	3.47	0.39
Girth of the lower shaft	21.46	2.5	0.37	<0.001	1.76	3.23	0.42
Girth of the head	19.75	1.73	0.22	<0.001	1.3	2.17	0.5
Girth of the neck	25.83	1.71	0.32	<0.001	1.06	2.35	0.30
Breadth of the neck	27.09	5.14	0.61	<0.001	3.93	6.36	0.53
MCB	27.91	2.91	0.52	<0.001	1.87	3.96	0.33
LCB	18.63	4.27	0.55	<0.001	3.18	5.37	0.49

p-Value and confidence limits show that the sample size is statistically significant.

Stature can be estimated using the formula Stature = MFL x 3.6, where MFL represents the maximum femoral length. Additionally, the formula y = a + bx is used to calculate the maximum femoral length, where y is the total length of the bone, a is the constant, b is the regression coefficient, and x is the fragment length. Simple linear regression equations were developed only for those parameters that demonstrated strong correlations, ensuring reliable and accurate stature estimation [14].

## Discussion

Stature estimation is a fundamental aspect of biological profiling in medico-legal inquiries. Regression analysis is the preferred method for determining relationships between long bone length, individual height, and fragment length. The mean total femur length in the present study (44.31 cm) was comparable to findings from other studies, which ranged from 42.01 cm to 44.90 cm [4,12,13,15]. Similarly, the mean bicondylar width or lower epiphyseal breadth (7.58 cm) aligned closely with previous findings, which reported values between 7.2 cm and 7.25 cm [12]. Proximal segment measurements were consistent with other South Indian studies [4], while the LCB and MCB measurements exhibited some variations when compared to earlier research [13].

The importance of femoral length in stature estimation has been confirmed by several recent studies. For instance, Bidmos demonstrated the utility of regression equations derived from femoral fragments in South African populations, highlighting the importance of population-specific models for accurate stature estimation [16]. Similarly, Mahakkanukrauh et al. emphasized the variability in skeletal proportions across ethnic groups and the need for tailored regression equations, particularly in Asian populations [17].

The observed differences in correlations between femoral length and its fragments among populations are attributed to genetic factors affecting skeletal growth and development across races [17]. Studies such as those by Raxter et al. have revised traditional methods of stature estimation, making them more applicable to modern populations by considering anatomical and methodological advancements [18]. These findings align with the results of the current study, which emphasize the importance of tailoring regression equations to the South Indian population.

While the present study does not differentiate between male and female femora, future research should address this limitation by incorporating sex differentiation and larger sample sizes. The inclusion of multiple parameters, as done in the present study, ensures that the regression equations are robust and applicable to a wide range of forensic scenarios.

## Conclusions

This study derived regression equations for determining the maximum femoral length from fragments in the South Indian population. All measurements showed positive correlations with total femur length, with shaft length exhibiting the strongest correlation. The derived equations can aid in stature estimation for forensic identification, disaster victim identification, medico-legal investigations, and anthropological research specific to the South Indian population.

The unique parameters used in this study allow for accurate stature estimation using any available part of the femur, enhancing its applicability in various forensic scenarios. Further research on larger sample sizes and diverse populations within India could refine these findings and contribute to a more comprehensive understanding of population-specific skeletal variations.
